# A witness seminar on the history of the Human Gene Mapping Workshops

**DOI:** 10.1016/j.gene.2016.02.030

**Published:** 2016-09-10

**Authors:** Emma M. Jones

**Affiliations:** History of Modern Biomedicine Research Group, School of History, Queen Mary University of London, Mile End Road, London E1 4NS, United Kingdom.

**Keywords:** HGMW, Human Gene Mapping Workshops, *MPI*, mannose phosphate isomerase

## Abstract

• Leading UK geneticists share behind-the-scenes insights from their careers.
• Recollections of early computing reveal struggles with technology.
• Problems with gene nomenclature are revisited: ‘their gene is like their baby’.

## 1 Introduction

In June 2015 the transcript of a ‘Witness Seminar’ on the history of the Human Gene Mapping Workshops c.1973–c.1991 was published by the History of Modern Biomedicine Research Group (www.histmodbiomed.org/witsem/vol54). The publication came from a meeting held in March 2014 to which key, mostly UK-based scientists contributed, two of whom had participated in the first Human Gene Mapping Workshop in 1973, and many of whom had been involved in subsequent HGMW (Table 1).

Witness Seminars are meetings to which a group of people who have been involved in particular discoveries, debates or events are invited to share their reminiscences and memories of ‘what really happened, the stories behind the published literature’ to quote Professor Tilli Tansey who heads the Group, and who developed this approach (Jones & Tansey, 2015).

Through the use of oral history in a group format, the Witness Seminar publications make a unique contribution to the international corpus of primary historical resources on twentieth century human/medical/clinical genetics, with a focus on the life stories of UK-based geneticists. The group format enables participants in the seminars to challenge, corroborate, and/or expand on the reminiscences of their peers. Although these Witness Seminar events and publications differ in style and content from the more traditional one-to-one approach to oral history, they also relate to the body of interviews conducted with scientists and clinicians by other individuals and institutions, such as: the Genetics and Medicine Historical Network of Cardiff University led by Professor Peter Harper (Genetics and Medicine Historical Network's, 2016); ‘Conversations in Genetics’ by the Genetics Society of America with USA-based scientists (Conversations in Genetics, 2016), and the National Human Genome Research Institute's staff interviews in Bethesda (NHGRI, 2016).

Similar to other high-quality oral history resources, the Witness Seminar publications are intended to interact with many other primary source materials which researchers and interested publics may engage with; for example, the ‘Codebreakers: Makers of Modern Genetics’ archival collections of geneticists and related organisations digitised by the Wellcome Library, London, in collaboration with other institutions (Codebreakers, 2016). The Witness Seminar publications also stand alone as significant historical narratives of biomedicine in their own right.

Each Witness Seminar meeting is facilitated by a chairman, the entire proceedings are recorded and transcribed, and then edited, with the addition of references, bibliographies and biographies, and published both in hard copy and as a freely available pdf. Several meetings have already been held and published on genetics-related or genetics-focused topics, including on haemophilia (Tansey & Christie, 1999); rhesus factor (Zallen et al., 2004); genetic testing (Christie & Tansey, 2003); clinical genetics (Harper et al., 2010); clinical cancer genetics (polyposis and familial colorectal cancer) (Jones & Tansey, 2013); and clinical molecular genetics (Jones & Tansey, 2014). All publications can be downloaded from the Group's website at: www.histmodbiomed.org/article/wellcome-witnesses-volumes.

## 2 Meeting report

**Fig. 1:**
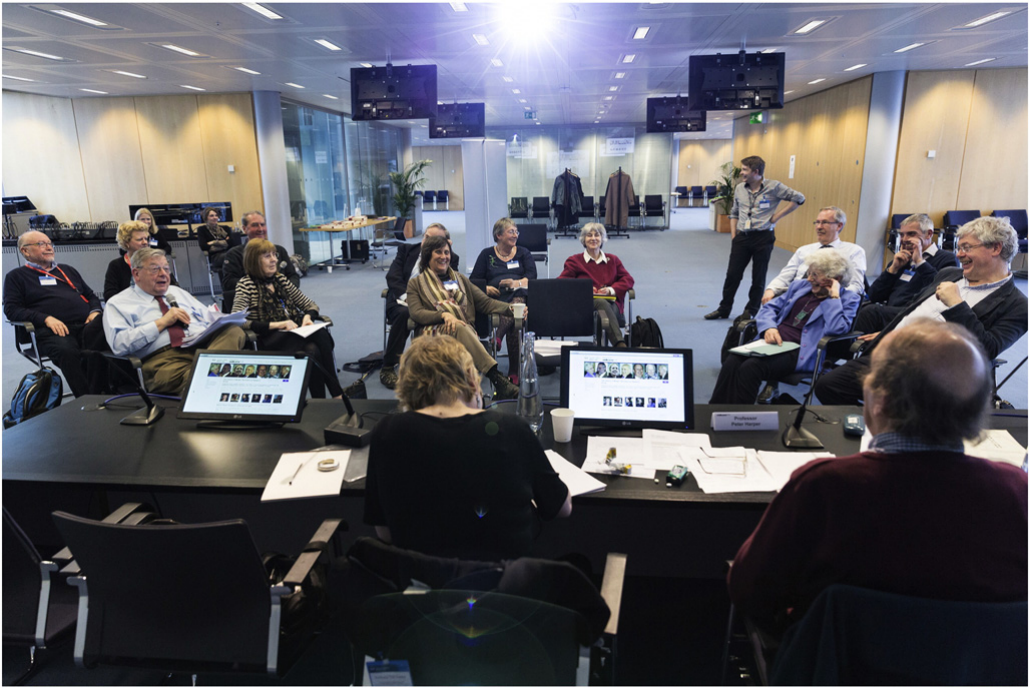
Participants at the Witness Seminar on Human Gene Mapping Workshops. Photograph copyright: 'Wellcome Library, London'.

Held over half a day, the Witness Seminar on the HGMW included a range of scientists and clinicians, as listed below, with Professor Peter Harper acting as a Chairman/facilitator (Fig. 1):

Professor Bert Bakker

(Technician in molecular genetics, 1977–1989/Head of the Laboratory for Diagnostic Genome Analysis, 1990–2015, Leiden University).

Professor Tim Bishop

(Assistant/Associate/Adjunct Professor at Department of Medical Informatics, University of Utah, 1979–1997; Director of the Leeds Institute of Cancer and Pathology, 2011–).

Professor Sir Walter Bodmer

(Professor of Genetics, University of Oxford, 1970–1979; Director of Research/Director General, Imperial Cancer Research Fund, 1979–1996; Head of the Cancer and Immunogenetics Laboratory, Weatherall Institute of Molecular Medicine, University of Oxford, 1996–2005).

Professor Ian Craig

(Demonstrator/Lecturer, Genetics Unit, 1970–1996/Professor in Genetics (titular), 1996–1998, Department of Biochemistry, University of Oxford; Head of Molecular Genetics 1998–2001/Professor of Molecular Psychiatric Genetics, King's College, London, 2001 — retired as Emeritus Professor 2015).

Professor Malcolm Ferguson-Smith

(Burton Professor of Medical Genetics, Glasgow University, 1973–1987; Professor/Head of Pathology, Cambridge University, then Professor of Pathology at Cambridge's Department of Veterinary Medicine, 1987–1998).

Professor Peter Harper

(Professor of Medical Genetics; University of Wales College of Medicine, 1971–2004; convenor of the Genetics and Medicine Historical Network, Cardiff University).

Professor Veronica van Heyningen

(Postdoctoral scientist, Medical Research Council's Human Genetics Unit, 1977–1992, then Head of the Cell and Molecular Genetics Section, 1992–2012).

Professor Maj Hultén

(Head of the Regional Genetics Service at East Birmingham/Heartlands Hospital, 1975–1997; Professor Emerita, Karolinska Institutet, Stockholm, 2012–).

Professor Sue Malcolm

(Died 2015. Emerita Professor of Molecular Genetics, University College London, Institute of Child Health).

Professor Michael Morgan

(Director of Research Partnerships and Ventures at the Wellcome Trust (WT)/Chief Executive of the Wellcome Trust Campus in Cambridge, retired from the WT in 2002).

Professor Sue Povey

(Chair of the HUGO Human Gene Nomenclature Committee, 1996–2007; Haldane Professor of Human Genetics, University College London, 2000–2007).

Professor Chris Rawlings

(Project Manager of bioinformatics for HGM10.5/11, Imperial Cancer Research Fund; Head of Department of Computational Systems Biology, Rothamsted Research, 2004–).

Professor Ellen Solomon

(Senior/Principal Scientist, Imperial Cancer Research Fund, 1976–1995; Head of the Department of Molecular Genetics, 1995–2009, Prince Philip Professor of Human Genetics, 2004–).

Dr. Susan Wallace

(Director, Americas Office of the Human Genome Organisation; Lecturer, Population and Public Health Sciences, University of Leicester).

**Fig. 2:**
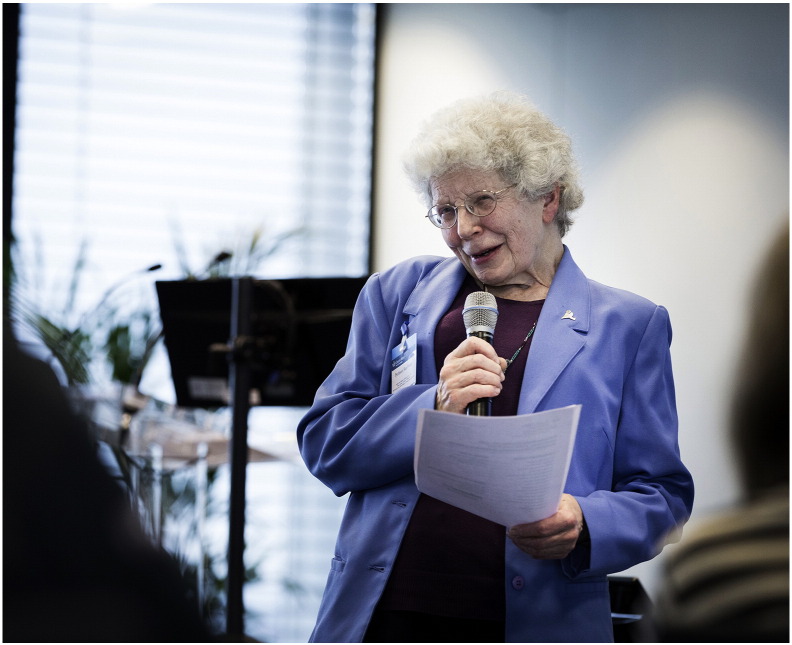
Professor Sue Povey. Photograph copyright: 'Wellcome Library, London'.

**Fig. 3:**
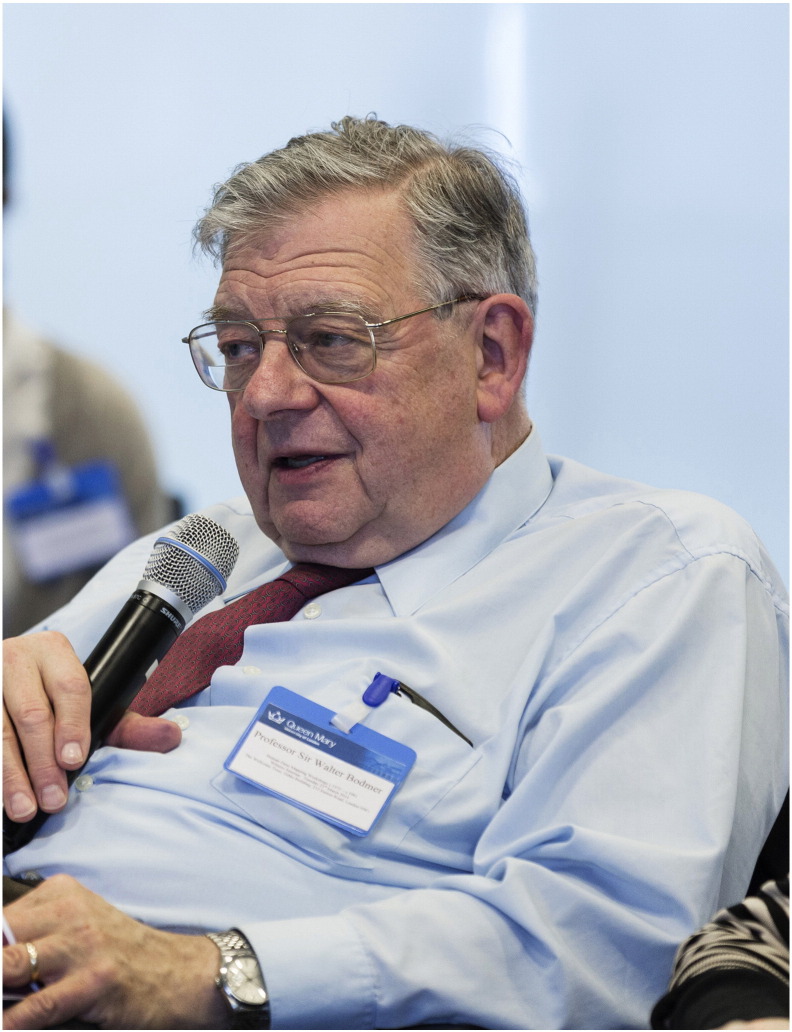
Professor Sir Walter Bodmer. Photograph copyright: 'Wellcome Library, London'.

Professor Sue Povey (Fig. 2) of the Galton Laboratory gave a historical introduction to the seminar, reviewing that Laboratory's contributions to human gene mapping research from its 1930s work on haemophilia and colour blindness, comparing it with research conducted elsewhere on *Drosophila* mapping. Later in the seminar she further framed the historical context for the breakthroughs brought by gene mapping thus: ‘In the 1970s, people, I anyway, didn't think we'd ever find a gene by where it was. I think that you (Walter Bodmer) introduced that in 1980, the reality of it’ (Jones & Tansey, 2015). Professor Sir Walter Bodmer (Fig. 3) outlined his own intellectual role in the field, while acknowledging the input of many others, and pointed to the role of somatic cell genetics research in paving the path to human gene mapping, with personal reference to his experience of working in Guido Pontecorvo's lab. On this trajectory, he also cited a particularly influential paper of the late 1960s: ‘…I think the initial, the first real, experiment that showed linkage mapping in hybrids was Weiss and Green, who used the hybrid technique but not with lymphocytes, and they investigated the chromosomal localization of the thymidine kinase marker that was ultimately shown to be on chromosome 17. That was really the first case that you could associate a marker with something you'd selected for …’ (Jones & Tansey, 2015).

Sir Walter's comments are illustrative of how he and other participants in the Witness Seminar filled in the historical ‘jigsaw’ of gene mapping research by linking their direct contributions to those of other geneticists.

On the first Human Gene Mapping Workshop, Professor Ian Craig discussed his experience of representing Bodmer's laboratory at HGM1 in Yale, 1973: ‘I have a strong recollection of arriving very nervously late at night at Yale and being introduced to a cocktail party … this very impressive, tall character, Frank Ruddle, came and said, “You have to talk first thing tomorrow morning”, which was not the best news’ (Jones & Tansey, 2015). Craig evidently survived his nerves, and recalled how the distinctive format for the Workshops emerged at that first event, and also some low-tech methods that were employed: ‘You could see John Edwards and Bette Robson going around and calculating lod scores on the backs of envelopes…’ (Jones & Tansey, 2015).

**Fig. 4:**
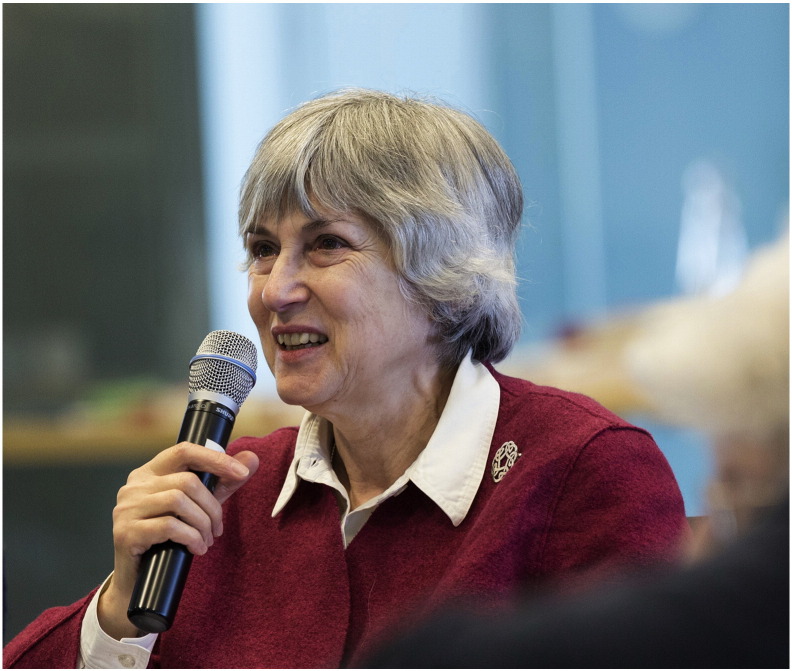
Professor Veronica van Heyningen. Photograph copyright: 'Wellcome Library, London'.

Originally from Sir Walter's laboratory, Professor Veronica van Heyningen (Fig. 4) attended HGM2 in Rotterdam as a post-doc, and also remembered being unnerved by the imposing Frank Ruddle – founder of Yale's Human Genetics Department and the convenor of HGM1 – when she disproved his assignment of mannose phosphate isomerase (*MPI*) to chromosome 7: ‘… we had assigned very firmly the mitochondrial malate dehydrogenase to chromosome 7 and it didn't co-segregate with *MPI* (Craig et al., 1974). Then, later on, we put *MPI* on a different chromosome, on 15.’ (van Heyningen et al., 1975) (Jones & Tansey, 2015).

**Fig. 5:**
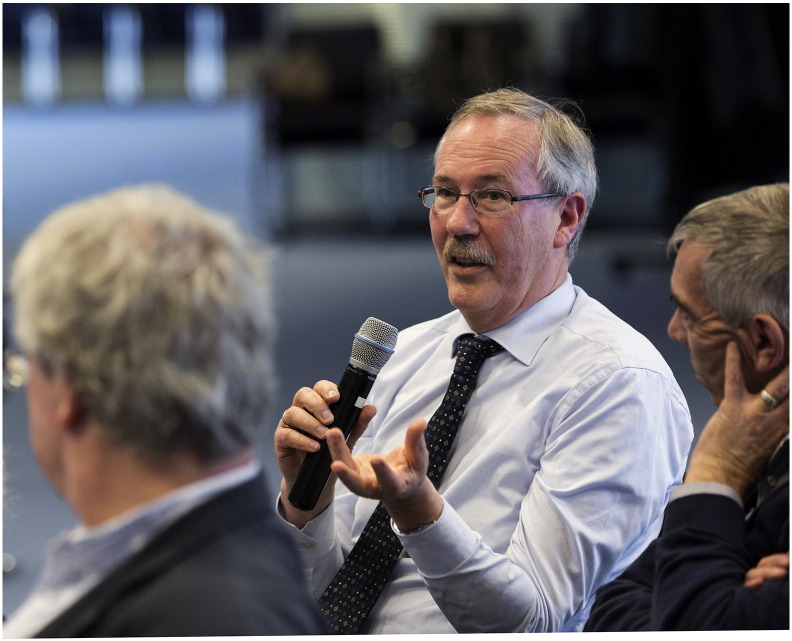
Professor Bert Bakker. Photograph copyright: 'Wellcome Library, London'.

The predominantly UK-based perspectives were broadened by Professor Bert Bakker from Leiden University (Fig. 5), who spoke of his own contributions and especially the importance of new techniques emerging in the 1970s for analysing chromosomes, such as heterochromatin staining, and the breakthrough of Y W Kan and A M Dozy's research on restriction fragment length polymorphisms by the end of that decade (Kan & Dozy, 1978). Despite improving techniques, Professor Malcolm Ferguson-Smith conveyed the painstaking work that his lab undertook on locating globin genes at the end of that decade: ‘… we knew which chromosomes the globin genes were on from the solution mapping …. However, their location on the chromosome was not known. But you (Professor Sue Malcolm) were able to show, doing those wretched silver grain counts, that beta globin mapped to the short arm of chromosome 11 and alpha globin to the short arm of chromosome 16’ (Jones & Tansey, 2015).

The seminar's participants discussed the wealth of data that had begun to proliferate by the late 1970s in terms of gene linkage analysis, and the subsequent influence of computing in the next decade of the Workshops' proceedings and documentation. In particular, Professor Chris Rawlings relived his role in coordinating the ambitious computational infrastructure for HGM10.5 at St John's College in Oxford in 1990, where the first version of the programme Genome Database was used: ‘…I often describe it a bit like running a rock concert, as you had to bring a team of roadies in to get all this stuff in place … there was a fair amount of blood, sweat and tears involved in this operation …’ (Jones & Tansey, 2015). Another novel insight into the gene mapping community concerned the sensitivities of nomenclature, when Professor Sue Povey recalled her time as Chair of the HUGO Human Gene Nomenclature Committee: ‘… criticism is so characteristic of nomenclature, it's always so controversial and people get so het up about it. Their gene is like their baby and everyone always agrees that one gene should only have one name but they are sure it should be theirs’ (Jones & Tansey, 2015). Also on politics, Professor Michael Morgan shared his experience of the rocky road towards funding the Human Genome Project (HGP) within the Wellcome Trust, and its decision ‘… that as soon as two kilobases of human DNA sequence was accumulated it would be released immediately on the internet, the “no intellectual property” position would be taken … That's had as much of an impact outside of genomics as probably the human genome has had within genomics’ (Jones & Tansey, 2015). Such candid behind-the-scenes revelations about the twists and turns of research into human genetics during the late twentieth century are precisely the stuff that our Witness Seminars are made of.

## 3 Conclusions

The Witness Seminar on Human Gene Mapping Workshops has created a significant addition to our outputs on the recent history of clinical and medical genetics. The resultant publication provides a unique resource for academics and other researchers/readers who are interested in progress towards the Human Genome Project, and current advances in genomics. We launched our volume at the European Society of Human Genetics satellite meeting, the 6th International Workshop on the History of Human Genetics, Glasgow, in June 2015, where we presented a poster on the seminar, and at which Professor Tansey gave the plenary lecture on ‘recording the voices of modern genetics’ (You're all history now, 2015). Since then, the homepage for the Human Gene Mapping Workshops c.1973–c.1991 publication has had over 250 unique page views on our Group's website, and we look forward to attracting future readers and researchers to this little-documented facet of the history of human genetics.

## Figures and Tables

**Table 1:** Locations and dates of the Human Gene Mapping Workshops.

HGM1	1973	New Haven
HGM2	1974	Rotterdam
HGM3	1975	Baltimore
HGM4	1977	Winnipeg
HGM5	1979	Edinburgh
HGM6	1981	Oslo
HGM7	1983	Los Angeles
HGM8	1985	Helsinki
HGM9	1987	Paris
HGM9.5	1988	New Haven
HGM10	1989	New Haven
HGM10.5	1990	Oxford
HGM11	1991	London
